# Influence of exogenously applied k-carrageenan at various concentrations on plant growth, phytochemical content, macronutrients, and essential oils of *Ocimum basilicum*

**DOI:** 10.1038/s41598-025-93479-3

**Published:** 2025-04-01

**Authors:** Mai Hosny Mohamed, Ahmed N. Abdelhamid, Mahmoud A. A. Ali, Basma T. Abd-Elhalim, Awaad M. Kandeel, Karim M. Hassan

**Affiliations:** 1 Department of Horticulture, Faculty of Agriculture, Ain Shams University, 68-Hadayek Shoubra, Cairo, 11241 Egypt; 2 Department of Agricultural Microbiology, Faculty of Agriculture, Ain Shams University, 68-Hadayek Shoubra, Cairo, 11241 Egypt

**Keywords:** Chlorophyll, Essential oils, K-carrageenan, Macroelements, *Ocimum basilicum*, Plant sciences, Secondary metabolism

## Abstract

Food safety and security are now among the most urgent problems to be resolved as the world’s population continues to grow. Intensive agriculture is required to meet the demands of a growing population and guarantee greater agricultural yield. Chemical pesticides and fertilizers are an essential part of intensive farming. Their extensive use accelerates the depletion of other important and minor nutrients, resulting in poor soil fertility and nutritional imbalance. There are serious health and environmental hazards associated with several of these hazardous agricultural chemicals. In context, for the first time, this study represents an innovative experiment exploring the impact of exogenously applied k-carrageenan on plant growth, physiological parameters, phytochemical content, macronutrients, and essential oil percentage in *Ocimum basilicum* plants. The investigation assessed the effect of varying k-carrageenan levels; 0.30, 0.60, 0.90, and 1.20 mM versus untreated control. The findings revealed that all k-carrageenan treatments significantly enhanced growth indicators compared to the control. The phytochemical analysis demonstrated that foliar application of k-carrageenan, particularly at 1.20 mM, significantly enhanced total chlorophyll, chlorophyll a, chlorophyll b, and total carbohydrate and essential oil percentage compared to the untreated control. *O. basilicum* essential oils show rich, nuanced flavors with higher levels of Methyl cinnamate, Camphor, trans-methyl cinnamate, Eucalyptol, Linalool, and β-Caryophyllene among treatments. Treatment effects were also observed in the macroelements content of Nitrogen (N), phosphorus (P), and potassium (K). k-carrageenan-induced alterations were noted in the contents of essential oil compounds. These results suggest that k-carrageenan can be a growth-promoting agent and significantly enhance essential oil yield, particularly in *O. basilicum* plants.

## Introduction

*Ocimum basilicum*, or basil, is a member of the Lamiaceae family, which also includes many other significant plants like lavender, rosemary, and mentha^1,2^. Compounds extracted from the plant have been employed in medicine, either in their natural form or after chemical modification^3^. *O. basilicum* exhibits extensive ethnomedicinal applications and its essential oil has demonstrated antiviral, larvicidal, antinociceptive, and antimicrobial properties^4–8^. Moreover, its essential oil is highly valued in the food industry for its flavoring and aromatic qualities, as well as its unique scent in perfumery^9,8,10^.

As a rich source of aromatic compounds, essential oil induces various biological processes, indicating its potential as a nematocidal, antibacterial, antifungal, antioxidant, and insect-repellent^3,10^. *O. basilicum* accessions have been categorized into different chemotypes based on their oil composition and geographical origin. The European type is characterized by methyl chavicol and linalool as the primary oil components, while most tropical chemotypes contain methyl cinnamate. Reunion *O. basilicum*, conversely, has a high concentration of methyl chavicol, while chemotypes in North Africa, Russia, Eastern Europe, and parts of Asia are rich in eugenol^11,12^.

In modern horticulture crop production, emphasis is placed on product quality, safety, and yield improvement. Enhancing the production of bioactive ingredients is particularly crucial in medicinal herbs, given the diverse biological functions of secondary metabolites^6^.

Carrageenans are sulfated linear polysaccharides isolated from various marine red algae^13^. These ingredients consist of a linear chain of D-galactose remains connected by glycosidic bonds replaced by ester sulphonic groups present in every two repeating galactose units. Carrageenans are categorized into three types based on sulfate groups that are present in each disaccharide unit: kappa (k)-carrageenan has 1, iota (k)-carrageenan has 2, and lambda (k)-carrageenan has 3 sulfate groups^14,15^.

Enzymes such as pyruvate dehydrogenase, glutamate dehydrogenase, inosine mono-phosphate dehydrogenase, isocitrate dehydrogenase, and glucose 6-phosphate dehydrogenase demonstrate increased activity in the presence of rubisco^16^. Furthermore, carrageenan serves as an elicitor to activate defense mechanisms against plant infections^16^. It primarily promotes plant development by controlling various metabolic pathways. Such as photosynthesis, cell division, purine and pyrimidine biosynthesis, and sulfur and nitrogen assimilation. Additionally, they trigger plant defensive responses against Abiotic stress like drought, salinity, and temperature^17–22^ by modulating several defense pathways, including ethylene, jasmonate, and salicylate signaling pathways^15,23,17^. Treatments with carrageenan have been shown to significantly increase plant height, trunk diameter, chlorophyll content, and net photosynthesis in various plant species^24,25,16^.

The present study aimed to determine the optimal treatment for enhancing plant development and phytochemical production as well as improving the quality and quantity of *O. basilicum* essential oils. While previous research has focused on increasing secondary metabolites in *O. basilicum*, this study is the first to investigate the influence of k-carrageenan on *O. basilicum* growth, phytochemical content, macronutrients, and essential oil. The research shows that k-carrageenan significantly improves *O. basilicum* growth parameters, nutrient content, and essential oil yield. It enhances plant health and productivity, particularly in culinary and medicinal contexts. The study also reveals that higher concentrations of k-carrageenan maximize the oil percentage in *O. basilicum*, benefiting industries relying on essential oils. k-carrageenan, a novel growth enhancer, offers innovative agricultural practices, enhancing crop yields and quality. It contributes to sustainable agriculture by improving plant growth and nutrient uptake, reducing chemical fertilizer use, and enhancing soil health. The study on k-carrageenan application in *O. basilicum* has identified gaps in its long-term effects, molecular understanding of its effects on plant growth and nutrient uptake, its broad applicability in agriculture, and its environmental impact, necessitating further research to understand its potential ecological consequences.

## Materials and methods

### Plant material and growing conditions

This study was carried out at the Ornamental and Medicinal Plants Farm, Department of Horticulture, Ain Shams University, Egypt. Uniform and healthy *O. basilicum* transplants, one-month-old and 15 cm in height, were sourced from a nursery in Giza, Egypt. The experimental design was a completely randomized design with 3 replicates. All pots (75 pots) were handed out by five k-carrageenan applications × 5 pots × 3 replicates. Each transplant was placed in a pot (0.35-m diameter) filled with a mixture of peat moss and sand (1:1) during the first week of March 2023. Irrigation was conducted regularly, 2 to 3 times per week. Fertilization was carried out using approximately half-strength Hoagland’s nutrition solution, applied once every ten days. After one month of cultivation, all experimental pots (75 pots) were located into five groups in the third week of April to receive exogenous application of k-carrageenan (Sigma Aldrich, St. Louis, MO, USA) at concentrations of zero (distilled water as a control), 0.30, 0.60, 0.90 and 1.20 mM. In the third week of May, the plants were harvested to examine their growth characteristics and plant chemical components.

### Measured parameters

When the plants achieved the best vegetative growth stage (at 75 days after planting) two pots from each replication were collected to assess the growth parameters. Shoot fresh weight of the whole plant (leaves and stem, g) was evaluated after sampling, while shoot dry weight (g) was identified by drying the samples in an air-forced ventilation oven (Binder FP056-230 V, Germany) at 105 °C. To determine the total content of nitrogen, phosphorus, and potassium, the plant samples were oven-dried at 70 °C and then wet-digested using a mix of H_2_SO_4_ and H_2_O_2_ as outlined by Cottenie et al.^26^. Total N content was estimated by the micro-Kjeldahl method utilizing 40% NaOH and 5% boric acid following AOAC^27^. Total K and P were estimated by employing ICP Mass Spectrometry (ICP-MS, China)^28^. Total carbohydrates were determined as g/100 g dry weight of herb as outlined by AOAC (2005)^29^. Chlorophyll-a and Chlorophyll-b (mg/g FW) were determined following Sumanta et al.^30^.

Steam distillation was utilized to separate the volatile oil from the dried vegetative parts of the *O. basilicum* plant, including the leaves and stem (both treated and untreated plants), employing a Clevenger glass apparatus. One hundred grams of the dried whole *O. basilicum* plant (leaves and stem) was separated in the steam distillation apparatus for 3 h. The *O. basilicum* oil was separated from the water and stored in dark glass bottles at 4 °C until the active ingredients were isolated and analyzed by Gas Chromatography-Mass Spectrometry (GC-MS) (GCMS 2400, USA). The oil percentage was calculated as grams of oil per 100 g of dry whole *O. basilicum* plant. After evaporation, the separated oil residue was thawed into 3 mL of ethyl acetate, and then 1 mL was transferred to a GC vial for GC/MS analysis. GC-MS was utilized to analyze the various components of *O. basilicum* volatile oil, including those present in modest quantities, as well as the main components of *O. basilicum* essential oil. Identification of components was based on a comparison of their mass spectra and retention times with those of authentic compounds, computer matching the WILEY and NIST libraries, and comparison of the fragmentation pattern of the mass spectral data with those reported in the literature. The analysis was conducted using a GC system (Agilent Technologies 7890 A) coupled with a mass-selective detector (MSD, Agilent 7000) and equipped with a polar Agilent HP-5ms (5%-phenyl methyl polysiloxane) capillary column (30 m × 0.25 mm i.d. and 0.25 μm film thickness). Helium was used as the carrier gas with a linear velocity of 1 mL/min. The injector and detector temperatures were set at 200 °C and 250 °C, in the same order, with a sample volume injected with 1 µL. The MS operating parameters were as follows: ionization potential 70 eV, interface temperature 250 °C, and acquisition mass range 50–800^31^.

### Statistical analysis

Statistical analyses were applied utilizing R software version 4.1.1 (https://www.r-project.org/). The Tukey’s range test at a significance level of *P* ≤ 0.05 was calculated to assess the significant differences among the studied treatments. The heatmap was generated using the Rcolor-Brewer package within the R software environment^32^.

## Results

### Growth parameters

**Fig. 1:**
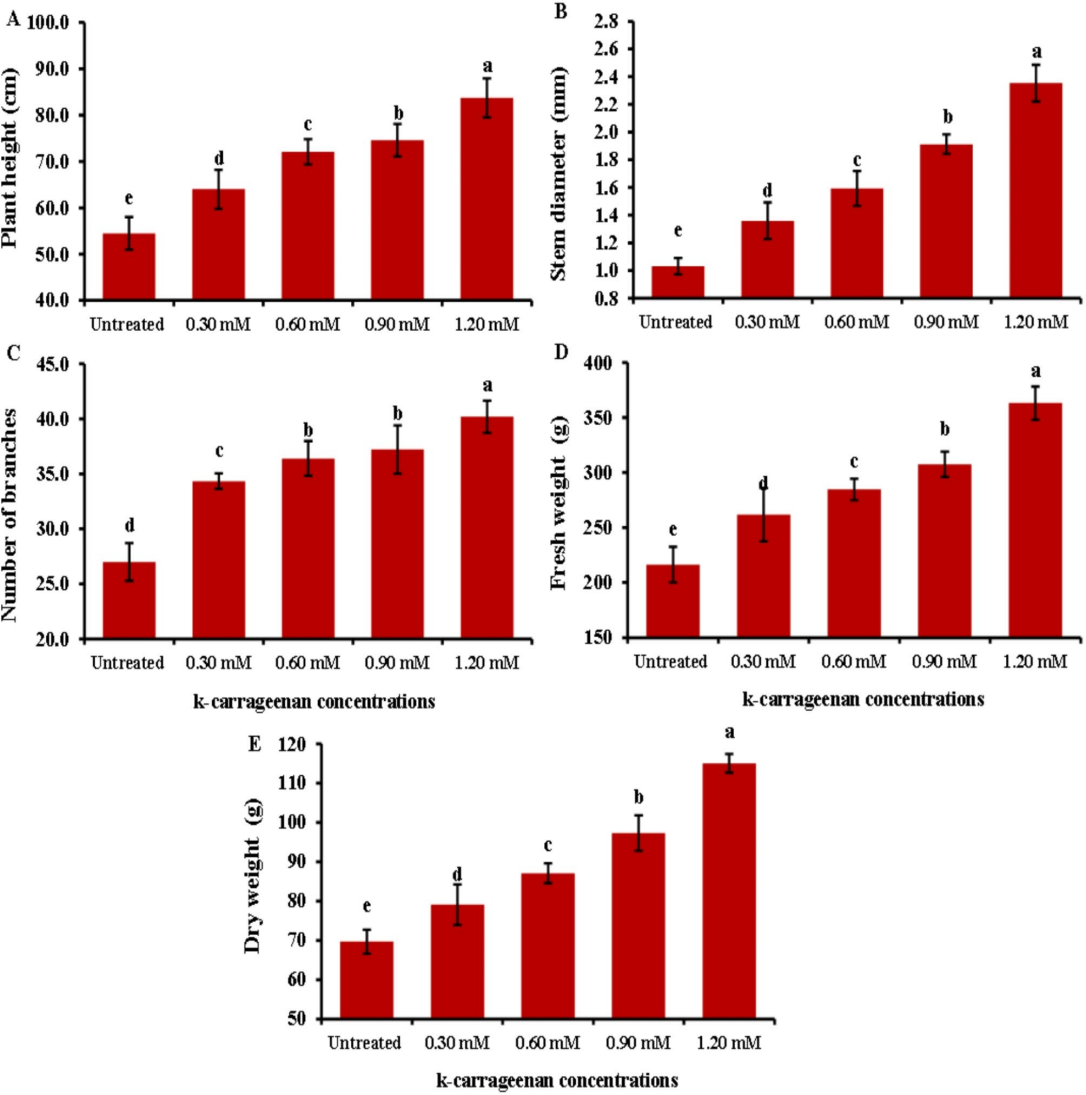
Influence of k-carrageenan treatment on plant height (**A**), Stem diameter (**B**), Number of branches (**C**), Fresh weight (**D**), and Dry weight (**E**) of *O. basilicum*. The bars represent standard deviation (SD) values, and different letters indicate significant differences, as determined by Tukey’s Studentized Range (HSD) Test (*p* < 0.05).

The impact of k-carrageenan on *O. basilicum* growth parameters is depicted in Fig. 1. The data suggest that the application of exogenous k-carrageenan led to an increase in all growth parameters (plant height, stem diameter, branch number, fresh weight, and dry weight) compared to untreated control plants. The lowest values of plant growth parameters were observed in the untreated control group, while the highest values were recorded in plants treated with 1.20 mM of k-carrageenan, with plant height reaching 83.73 cm, stem diameter measuring 2.35 cm, branch number totaling 40.20, fresh weight amounting to 363.07 g, and dry weight reaching 115.0 g compared to the other concentrations tested.

### Chlorophyll A, B, total chlorophyll, and total carbohydrates

**Fig. 2:**
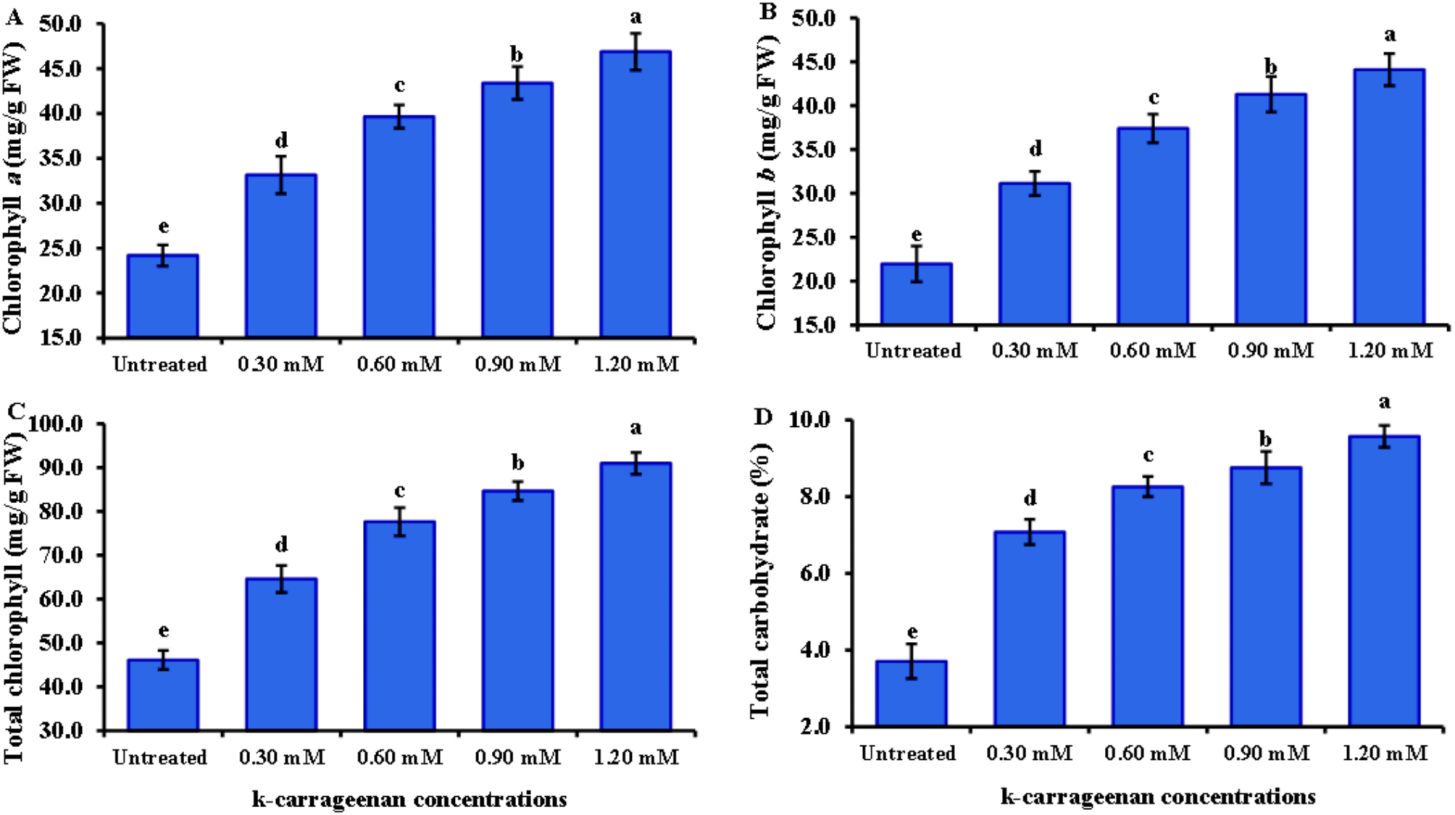
Influence of k-carrageenan treatment on chlorophyll a (**A**), Chlorophyll b (**B**), Chlorophyll a (**C**), Total chlorophyll (**D**), and Total carbohydrate (**E**) of *O. basilicum*. The bars represent standard deviation (SD) values, and different letters indicate significant differences, as determined by Tukey’s Studentized Range (HSD) Test (*p* < 0.05).

The results of chlorophyll a, b, total chlorophyll, and total carbohydrates are presented in Fig. 2. The application of k-carrageenan led to a significant enhancement in chlorophyll a, b, and total chlorophyll. The lowest values of 24.20, 21.95, and 46.15 were observed under untreated control conditions, respectively. Conversely, the data revealed that treatment with 1.20 mM k-carrageenan recorded the maximum values of 46.89, 44.09, and 90.98 for chlorophyll a, b, and total chlorophyll, respectively. Total carbohydrates were reduced in nontreated plants, while they increased and reached the highest value of 9.57 with the highest level of k-carrageenan.

### Content of macroelements

**Fig. 3:**
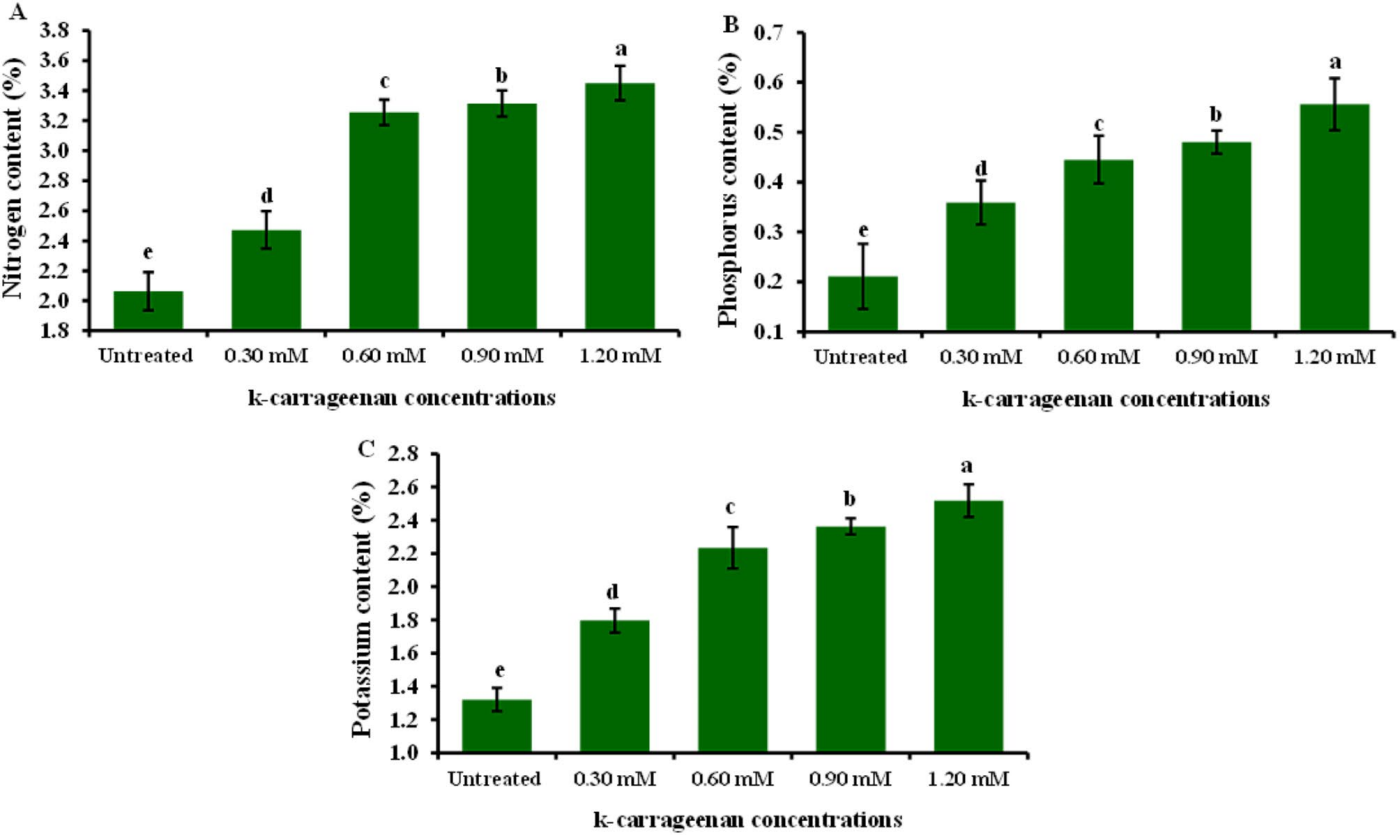
Influence of k-carrageenan treatment on content of Nitrogen (**A**), Phosphorus (**B**), and Potassium (**C**) of *O. basilicum*. The bars represent standard deviation (SD) values, and different letters indicate significant differences, as determined by Tukey’s Studentized Range (HSD) Test (*p* < 0.05).

The N, P, and K content in *O. basilicum* was influenced by k-carrageenan, as depicted in Fig. 3. Overall, the application of k-carrageenan led to a significant increase in the levels of N, P, and K. Moreover, escalating the concentration of k-carrageenan from 0.30 to 1.20 mM resulted in a significant rise in the percentages of N, P, and K. The highest percentages of N, P, and K were noticed with the application of 1.20 mM k-carrageenan (3.45, 0.56, and 2.52%, respectively), compared to all other concentrations and untreated control.

### Oil percentage

**Fig. 4:**
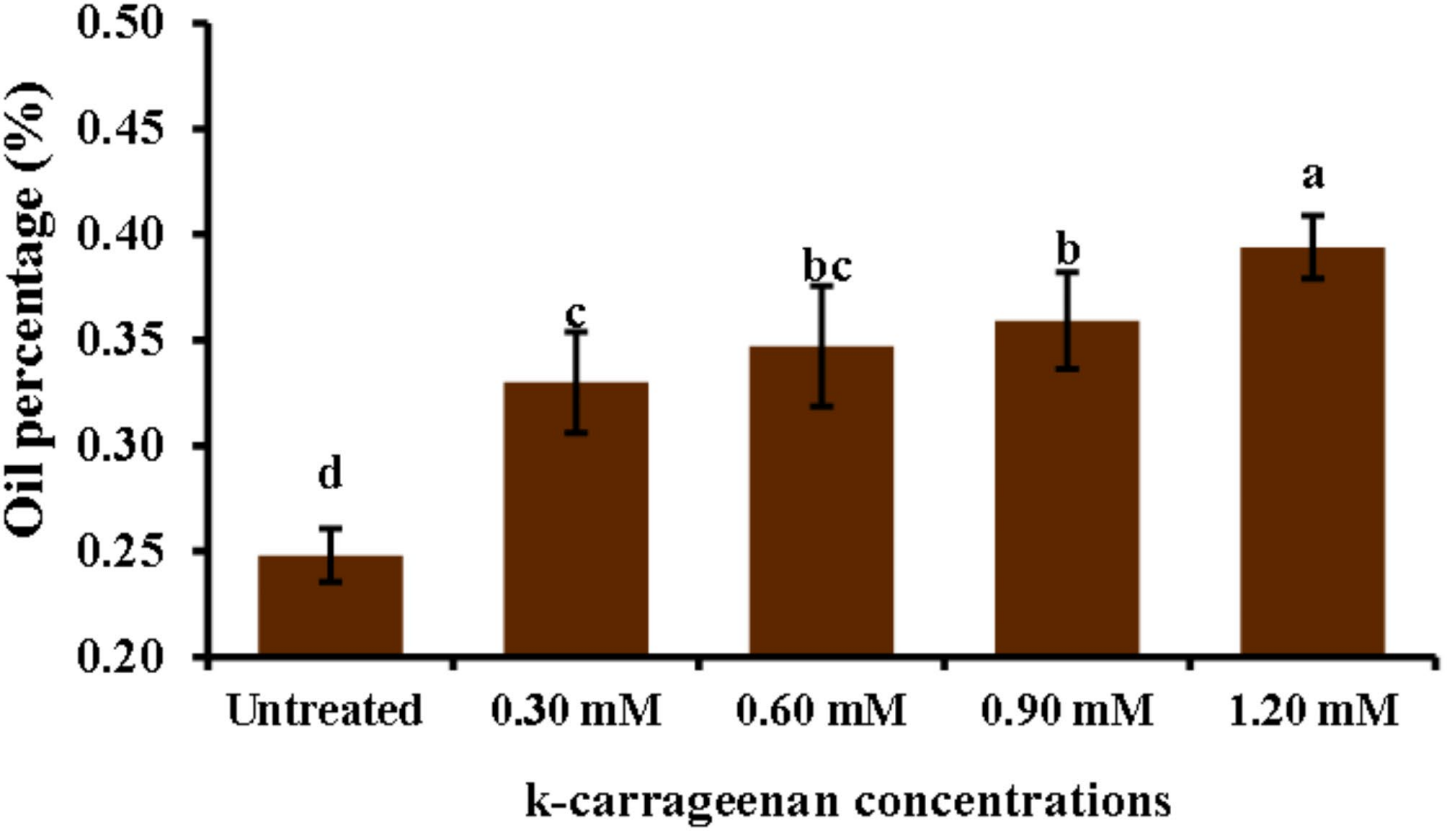
Influence of k-carrageenan treatment on yield of *O. basilicum* essential oil (%) in the dried vegetative parts (leaves and stem). The bars represent standard deviation (SD) values, and different letters indicate significant differences, as determined by Tukey’s Studentized Range (HSD) Test (*p* < 0.05).

The results illustrated in Fig. 4 showed that the oil percentage was at its minimum under untreated control. However, the oil percentage reached its maximum (0.39%) at a high level of k-carrageenan 1.20 mM followed by 0.90 and 0.60 mM.

### Association among applied k-carrageenan concentrations and evaluated parameters

**Fig. 5:**
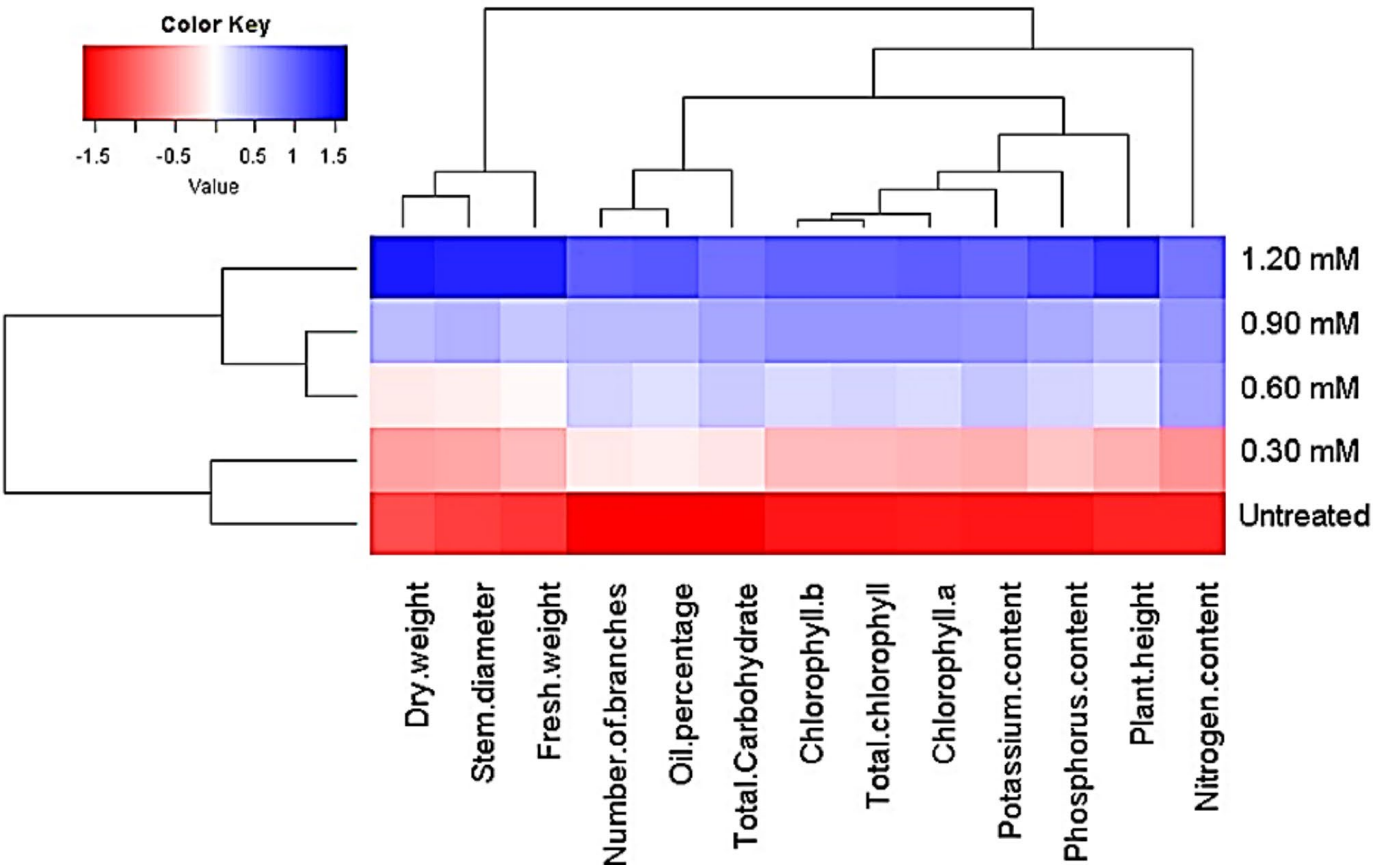
Heatmap for the evaluated parameters of *O. basilicum* plants treated with different concentrations of k-carrageenan. Parameters. Red color and blue color reveal high and low values for the corresponding parameters, respectively.

Exploring the association between applied treatments and the parameters studied is pivotal for providing valuable insights. Utilizing the heatmap and hierarchical clustering, studied parameters including plant growth, phytochemical content, macronutrients, and essential oil percentage of *O. basilicum* plants, grouped the applied treatments into distinct clusters (Fig. 5). It was observed that the applied k-carrageenan with a concentration of 1.20 mM, followed by 0.90, 0.60, and 0.30 mM, demonstrated favorable performance across all evaluated parameters. Conversely, the untreated control exhibited unfavorable performance (Fig. 5).

### Impact of k-carrageenan on *O. basilicum* essential oils

**Fig. 6:**
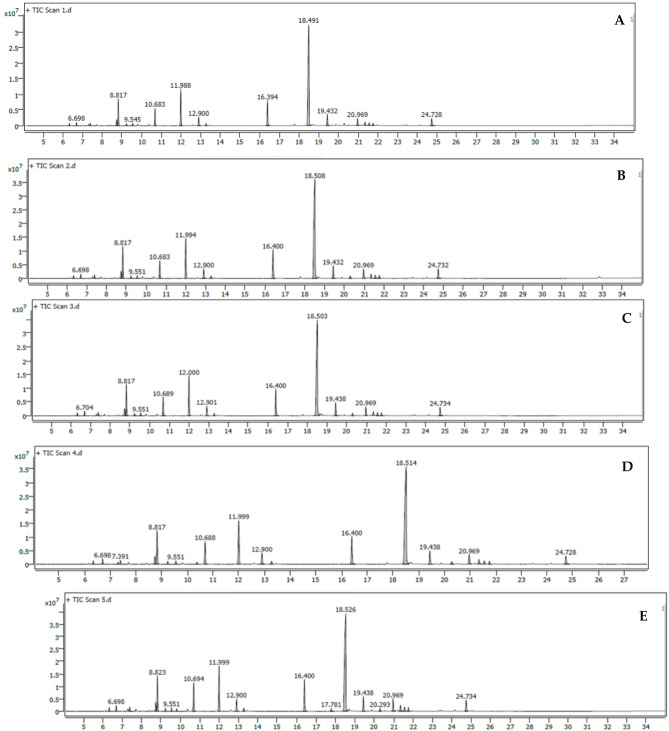
GC-MS Chromatograms of *O. basilicum* volatile oils. (**A**) Untreated control, (**B**) *O. basilicum* treated with 0.30 mM k-carrageenan, (**C**) *O. basilicum* treated with 0.60 mM k-carrageenan, (**D**) *O. basilicum* treated with 0.90 mM k-carrageenan and (**E**) *O. basilicum* treated with 1.20 mM k-carrageenan.

The application of k-carrageenan influenced the content of essential oils in *O. basilicum* plants. Table 1 presents the results of GC-MS analysis of essential oils. Following cultivation under various concentrations of k-carrageenan, 24 chemical compounds were identified in *O. basilicum*. These compounds were detected across different k-carrageenan concentrations, whereas the lowest number of essential oil compounds (19) was observed in the untreated plants with k-carrageenan (Fig. 6). Additionally, the data revealed a significant and noticeable increase in the percentage of essential oil in treated plants with k-carrageenan at all concentrations (0.30, 0.60, 0.90, and 1.20 mM) compared to untreated control. k-carrageenan treatments led to elevated values of essential oil components compared to the control. Compounds such as α-Pinene, Camphene, β-Pinene, D-Limonene, Eucalyptol, β-Ocimene, γ-Terpinene, Linalool, (+)-Camphor, Terpinen-4-ol, α-Terpineol, trans-Methyl cinnamate, Methyl cinnamate, β-Caryophyllene, β-Copaene, Bicyclogermacrene, δ-Guaiene, γ-Cadinene, and Tau-Cadinol acetate were detected in plants both with and without k-carrageenan treatments. Sabinene and terpinolene were observed in plants treated with 0.90 mM of k-carrageenan, while trans-Sabinene hydrate and Eugenol were detected in plants treated with 1.20 mM of k-carrageenan. Humulene was recorded only in the treated plants with k-carrageenan (Table 1).

**Table 1:** Influence of k-carrageenan treatments on oil component**s** in *O. basilicum* plants.

Peak	RT	Component name	K-carrageenan concentrations				
			Untreated control	0.30 mM	0.60 mM	0.90 mM	1.20 mM
1	6.334	α-Pinene	0.53	0.59	0.64	0.66	0.65
2	6.698	Camphene	0.85	0.95	1.07	1.06	1.04
3	7.298	Sabinene				0.40	
4	7.391	β-Pinene	0.68	0.82	0.83	0.83	0.79
5	8.730	D-Limonene	1.55	1.73	1.83	1.81	1.79
6	8.817	Eucalyptol	7.44	7.8	7.89	7.8	7.16
7	9.233	β-Ocimene	0.53	0.59	0.54	0.65	0.58
8	9.545	γ-Terpinene	0.67	0.66	0.7	0.76	0.71
9	9.811	Trans-Sabinene hydrate					0.53
10	10.371	Terpinolene				0.47	
11	10.683	Linalool	4.84	4.32	4.84	5.33	6.18
12	11.988	(+)-Camphor	11.28	10.95	11.54	11.56	10.63
13	12.900	Terpinen-4-ol	2.58	2.43	2.56	2.78	2.69
14	13.270	α-Terpineol	0.65	0.69	0.69	0.73	0.70
15	16.394	Trans-Methyl cinnamate	7.77	7.92	7.77	7.4	7.50
16	17.781	Eugenol					0.67
17	18.491	Methyl cinnamate	49.9	48.36	46.76	46.27	45.24
18	19.432	β-Caryophyllene	3.38	3.44	3.63	3.38	3.34
19	20.290	Humulene		0.68	0.74	0.66	0.69
20	20.969	β-Copaene	2.26	2.38	2.4	2.41	2.84
21	21.344	Bicyclogermacrene	1.08	1.15	1.18	1.24	1.56
22	21.558	δ-Guaiene	0.88	0.96	0.97	0.88	1.08
23	21.754	γ-Cadinene	0.77	0.83	0.83	0.73	0.87
24	24.728	Tau-Cadinol acetate	2.35	2.76	2.58	2.2	2.74

## Discussion

This study reported for the first time the usage of k-carrageenan to promote the biosynthesis of active ingredient substances in *O. basilicum*. *O. basilicum* plants treated with k-carrageenan exhibited a significant increase in all growth parameters; plant height, number of branches, stem diameter, and fresh and dry weight, compared to untreated plants. Overall, the application of 1.20 mM k-carrageenan resulted in substantial improvements across all growth parameters compared to the untreated control group by 1.5, 2.35, 1.48, 1.73, and 1.64-folds increases for plant height, stem diameter, total branch number, fresh weight, and dry weight, respectively. All applied k-carrageenan concentrations resulted in a significant enhancement in chlorophyll a, b, total chlorophyll, and total carbohydrate content in *O. basilicum* plants compared to untreated plants by 1.94, 2.0, 1.97, and 2.5, respectively. In addition, N, P, and K were noticed to increase by 3.45, 0.56, and 2.52% with 1.46, 2.8, and 1.94-folds, respectively compared to control plants. This suggests that k-carrageenan acts as an effective biostimulant, promoting better growth, structural integrity, and biomass accumulation in *O. basilicum* plants. The untreated control group consistently showed the lowest values across all parameters, highlighting the beneficial effects of k-carrageenan treatment. This may be attributed to the high sulfur content in k-carrageenan. Sulfur plays a crucial function in plant metabolism as it is required for the synthesis of plant proteins, vitamins, phytochelatins, thiamine, phenolic compounds, flavonoids, coenzyme A, glutathione, amino acids, and enzymes as well as its necessity for chlorophyll synthesis, which enhances the photosynthesis process, leading to increased carbohydrate accumulation^33–36^. Sulfur also stimulates nodulation in legumes and aids in the development and activation of specific enzymes and vitamins, ultimately contributing to increased plant growth and productivity. Since sulfur is water-soluble, it is readily absorbed by plant leaves from k-carrageenan extract, which is soluble^34^. This finding aligns with previous studies by Naeem et al.^37^, who noticed that spraying k-carrageenan increased productivity in *Catharanthus roseus* and significantly increased its performance. Also, the enhancement in leaf area resulting from k-carrageenan treatment enhanced solar harvesting, carbon dioxide (CO_2_) consumption, and chlorophyll content, thereby improving photosynthesis rates and leading to the accumulation of plant dry matter. The k-carrageenan application significantly boosted nitrogen, phosphorus, and potassium levels in *O. basilicum* plants. Increasing the concentration from 0.30 to 1.20 mM led to higher macroelements percentage, with the highest values at 1.20 mM being 3.45% nitrogen, 0.56% phosphorus, and 2.52% potassium, exceeding all other concentrations and the untreated control. El-Beltagi et al.^38^ reported that the use of k-carrageenan at concentrations of 400, 600, and 800 ppm resulted in a substantial increase in all growth parameters, including plant height, number of branches, and stem diameter. Furthermore, Mousavi et al.^39^ found that treatments with 1 g/L k-carrageenan increased *O. basilicum* shoot length and leaf area. This treatment stimulated *O. basilicum* growth by enhancing shoot length and leaf area, as well as increasing phenylalanine ammonia-lyase activity, phenolic ingredients, lignin concentrations, and antioxidant activity. Consequently, k-carrageenan can be considered to activate the phenylpropanoid pathway due to its high sulfur content, which is a type of linear sulfated polysaccharide^40^. Sulfur interacts with nearly every major macronutrient, secondary nutrient, and micronutrient, altering nutrient uptake and usage, which can impact crop growth and production. Sulfur and nitrogen are strongly associated, with sulfur increasing the efficiency of plant nitrogen absorption. Sulfur’s involvement in nitrate conversion to amino acids also links it to nitrogen. Crops with high nitrogen requirements usually have high sulfur requirements^33^. Sulfur and phosphorus are both essential nutrients absorbed by plants from the soil in their anionic state, and plants require equivalent amounts of these components^41^. The role of sulfur and potassium in increasing agricultural output and increasing crop quality is well-established^42,43^, with increased potassium absorption resulting in more blooms in plants. The oil percentage in *O. basilicum* was lowest in the untreated control and reached a maximum of 0.39% with 1.20 mM k-carrageenan, with 1.56- a 1.56-fold increase compared to the control treatment. The quantity and quality of essential oils components in plants can be affected by various environmental factors and nutrition. For instance, it has been reported that the essential oil of *O. basilicum* plants changes with nutrition^44^. k-carrageenan is an important biostimulant that affects essential oil biosynthesis, with enhanced essential oils content^45,46^. The use of k-carrageenan significantly increased the essential oil output and the content of the key ingredients of the essential oils compared to the control observed in the fennel study^45^. Essential oils of *O. basilicum* exhibited nuanced and rich flavor notes, with higher levels of Methyl cinnamate, representing between 45.24 and 49.9% in the oil among treatments. Intermediate proportions of Camphor (10.63 to 11.56%), trans-methyl cinnamate (7.4 to 7.92%), Eucalyptol (7.16 to 7.89%), Linalool (4.32 to 6.18%), and β-Caryophyllene (3.34 to 3.63%) were also observed among all treatments. The presence of Methyl cinnamate as the main component in our species is consistent with previous research on different *O. basilicum* species^1,47^. Camphor was detected in this study at the highest level under 0.30 mM k-carrageenan, as reported in previous studies^1,48^. Some compounds were not found in the untreated plants but appeared under k-carrageenan treatment. Sabinene and Terpinolene were recorded in plants treated with 0.60 mM k-carrageenan compared to other treatments. Additionally, Sabinene^49^ and Terpinolene^50^ have been reported in *O. basilicum*. k-carrageenan at 0.90 mM enhanced more compounds compared to the control, such as Tran sabinene hydrate^51^ and Eugenol^52^. Furthermore, Humulene was observed in k-carrageenan-treated plants^53^. The essential oil percentage increased with k-carrageenan application due to a significant increase in dry and fresh matter yields. A similar effect was found in Mungbam^54^ and peachy^55^, as well as an increase in essential oils in fennel^45^.

## Conclusions

The investigation into the effects of exogenously applied k-carrageenan on *O. basilicum* has yielded significant insights into its potential as a growth-promoting agent. The study demonstrates that varying concentrations of k-carrageenan can enhance key growth parameters, improve phytochemical content, and increase essential oil yield in basil plants. These findings underscore the importance of k-carrageenan not only as a biostimulant that can lead to higher agricultural productivity but also as a sustainable alternative to synthetic fertilizers. The research highlights the potential economic benefits for farmers, particularly in the context of increasing market demand for high-quality and organic herbs. Additionally, it opens avenues for further exploration into the mechanisms by which k-carrageenan influences plant physiology, nutrient uptake, and overall health. Addressing existing research gaps, such as the long-term effects and broader applicability of k-carrageenan across different crops, will be essential for developing comprehensive agricultural practices that are both effective and sustainable. In conclusion, this study contributes valuable knowledge to the field of horticulture and agricultural science, advocating for the integration of natural biostimulants like k-carrageenan in modern farming practices. As the agricultural sector continues to face challenges related to food security and environmental sustainability, research of this nature is crucial for fostering innovative solutions that benefit both farmers and the ecosystem.

## Data Availability

Data sets generated during the current study are available from the corresponding author upon reasonable request.

## References

[1] Telci, I., Bayram, E., Yilmaz, G. & Avci, B. Variability in essential oil composition of Turkish Basils (*Ocimum Basilicum* L). *Biochem. Syst. Ecol.* **34** (6), 489–497. 10.1016/j.bse.2006.01.009 (2006).

[2] El-Sayed, S. M. et al. Exogenous Paclobutrazol reinforces the antioxidant and antimicrobial properties of lavender (*Lavandula officinalis* L.) oil through modulating its composition of oxygenated terpenes. *Plants* **11** (12), 1607. 10.3390/plants11121607 (2022). 35736758 10.3390/plants11121607 PMC9230930

[3] Labra, M. et al. Morphological characterization, essential oil composition and DNA genotyping of *Ocimum basilicum* L. cultivars. *Plant. Sci.* **167** (4), 725–731. 10.1016/J.PLANTSCI.2004.04.026 (2004).

[4] Vieira, R. F. & Simon, J. E. Chemical characterization of basil (*Ocimum* spp.) based on volatile oils.* Flavour Fragr. J.*** 21** (2), 214–222. 10.1002/ffj.1513 (2006).

[5] Li, Q. X. & Chang, C. L. Basil (*Ocimum basilicum* L.) oils. In* Essential Oils in Food Preservation, Flavor and Safety* 231–238. 10.1016/B978-0-12-416641-7.00025-0 (2016).

[6] Husen, A. & Herbs *Shrubs and Trees of Potential Medicinal Benefits* (CRC, 2022). 10.1201/9781003205067

[7] Bachheti, A. J., Bhalla, P., Bachheti, R. K. & Husen, A. Growth and development of medicinal plants, and production of secondary metabolites under Ozone pollution. In: (ed Husen, A.) Environmental Pollution and Medicinal Plants. CRC, Boca Raton, 25–45. 10.1201/9781003178866-2 (2022).

[8] Elansary, H. O., Yessoufou, K., Shokralla, S., Mahmoud, E. A. & Skalicka-Woźniak, K. Enhancing mint and Basil oil composition and antibacterial activity using seaweed extracts. *Ind. Crops Prod.* **92**, 50–56. 10.1016/j.indcrop.2016.07.048 (2016).

[9] Kashyap, C. P., Ranjeet, K., Vikrant, A. & Vipin, K. Therapeutic potency of *Ocimum Kilimandscharicum Guerke*—a review. *Glob J. Pharmacol.* **5** (3), 191–200 (2011).

[10] Shafique, M., Khan, J. S. & Khan, H. N. Study of antioxidant and antimicrobial activity of sweet Basil (*Ocimum Basilicum*) essential oil. *Pharmacol. Online* **1**, 105–111 (2012).

[11] Hanif, A. M. et al. Essential oil composition, antimicrobial and antioxidant activities of unexplored Omani Basil. *J. Med. Plants Res.* **5** (5), 751–757 (2011).

[12] Saggiorato, A. G. et al. Antifungal activity of Basil essential oil (*Ocimum Basilicum* L.): evaluation in vitro and on an Italian-type sausage surface. *Food Bioprocess. Technol.* **5** (1), 378–384. 10.1007/s11947-009-0310-z (2012).

[13] Sangha, J. S. et al. Carrageenans, sulphated polysaccharides of red seaweeds, differentially affect Arabidopsis thaliana resistance to *Trichoplusia ni* (Cabbage looper). *PLoS One* **6**, e26834. 10.1371/journal.pone.0026834 (2011). 22046375 10.1371/journal.pone.0026834 PMC3203909

[14] González, A., Castro, J., Vera, J. & Moenne, A. Seaweed oligosaccharides stimulate plant growth by enhancing carbon and nitrogen assimilation, basal metabolism, and cell division. *J. Plant. Growth Regul.* **32**, 443–448. 10.1007/s00344-012-9309-1 (2013).

[15] Zia, K. M. et al. A review on synthesis, properties and applications of natural polymer based Carrageenan blends and composites. *Int. J. Biol. Macromol.* **96**, 282–301. 10.1016/j.ijbiomac.2016.11.095 (2017). 27914965 10.1016/j.ijbiomac.2016.11.095

[16] Castro, J., Vera, J., González, A. & Moenne, A. Oligo-carrageenans stimulate growth by enhancing photosynthesis, basal metabolism, and cell cycle in tobacco plants. *Burley) J. Plant. Growth Regul.* **31** (2), 173–185. 10.1007/S00344-011-9229-5/FIGURES/6 (2012).

[17] Prajapati, V. D., Maheriya, P. M., Jani, G. K., Solanki, H. K. & Retracted Carrageenan: A natural seaweed polysaccharide and its applications. *Carbohydr. Polym.* **105**, 97–112. 10.1016/j.carbpol.2014.01.067 (2014). 24708958 10.1016/j.carbpol.2014.01.067

[18] Ali, M. A. A. et al. Melatonin as a key factor for regulating and relieving abiotic stresses in harmony with phytohormones in horticultural plants—a review. *J. Soil. Sci. Plant. Nutr.* 1–21. 10.1007/s42729-023-01586-9 (2023).

[19] Lasheen, F. F. et al. Exogenous application of humic acid mitigates salinity stress on pittosporum (*Pittosporum tobira*) plant by adjusting the osmolytes and nutrient homeostasis. *J. Crop Health* **76** (1), 317–325. 10.1007/s10343-023-00939-9 (2024).

[20] de Medeiros, R. L. S., de Paula, R. C. & de Souza, J. V. O. Abiotic stress on seed germination and plant growth of *Zeyheria tuberculosa*. *J. Res.* **34**, 1511–1522. 10.1007/s11676-023-01608-3 (2023).

[21] Hassan, K. M. et al. Silicon: a powerful aid for medicinal and aromatic plants against abiotic and biotic stresses for sustainable agriculture. *Horticulturae* **10** (8), 806. 10.3390/horticulturae10080806 (2024).

[22] Tawfik, E. et al. Molecular identification of zantedeschia culture with determination of its morphometric and metabolic activities for mediterranean acclimatization. *Plants* **11** (17), 2311. 10.3390/plants11172311 (2022). 36079693 10.3390/plants11172311 PMC9460599

[23] El-Beltagi, H. S. et al. Phytochemical and potential properties of seaweeds and their recent applications: a review. *Mar. Drugs* **20**, 342. 10.3390/md20060342 (2022). 35736145 10.3390/md20060342 PMC9227187

[24] González, A., Contreras, R. A. & Moenne, A. Oligo-carrageenans enhance growth and contents of cellulose, essential oils and polyphenolic compounds in *Eucalyptus globulus* trees. *Molecules* **18**, 8740–8751. 10.3390/molecules18088740 (2013). 23887716 10.3390/molecules18088740 PMC6270522

[25] Bi, F., Iqbal, S., Arman, M., Ali, A., Hassan, M. U. & Carrageenan as an elicitor of induced secondary metabolites and its effects on various growth characters of Chickpea and maize plants. *J. Saudi Chem. Soc.* **15**, 269–273. 10.1016/j.jscs.2010.10.003 (2011).

[26] Cottenie, A., Verloo, M., Kiekens, L., Velgh, G. & Camerlynech, R. *Chemical Analysis of Plants and Soils*, vol. 42, 80–284 (Laboratory of Analytical Agrochemistry State University, 1982).

[27] A.O.A.C. *Official Method of Analysis of Association of Official Analytical Chemists* 12th edn (Association of Official Analytical Chemists, 1975).

[28] Benton, J. J. *Laboratory Guide for Conducting Soil Test and Plant Analysis* (CRC, 2001).

[29] A.O.A.C. Official methods of analysis of the association of official analytical chemists, 12th edn. (2005).

[30] Sumanta, N., Haque, C. I., Nishika, J. & Suprakash, R. Spectrophotometric analysis of chlorophylls and carotenoids from commonly grown fern species by using various extracting solvents. *Res. J. Chem. Sci.* **4**, 63–69 (2014).

[31] Santana, P. M. et al. Gas chromatography-mass spectrometry study from the leaves fractions obtained of *Vernonanthura patens* (Kunth) H. Rob. *Int. J. Organ. Chem.* **3**, 105–109. 10.4236/ijoc.2013.32011 (2013).

[32] SAS. *SAS/STAT User’s Guide, Release 6.03 Ed* (SAS Institute Inc., 1988).

[33] Stewart, W. M. Sulfur-The 4th Major Nutrient, IPNI Plant Nutrition TODAY, Spring, 7 (2010).

[34] Narayan, O. P., Kumar, P., Yadav, B., Dua, M. & Johri, A. K. Sulfur nutrition and its role in plant growth and development. *Plant. Signal. Behav. e2030082*. 10.1080/15592324.2022.2030082 (2022). 10.1080/15592324.2022.2030082 PMC10730164 35129079

[35] Leustek, T., Martin, M. N., Bick, J. A. & Davies, J. P. Pathways and regulation of sulfur metabolism revealed through molecular and genetic studies. *Annu. Rev. Plant. Physiol. Plant. Mol. Biol.* **51**, 141–165. 10.1146/annurev.arplant.51.1.141 (2000). 15012189 10.1146/annurev.arplant.51.1.141

[36] Lewandowska, M. & Sirko, A. Recent advances in Understanding plant response to sulfur-deficiency stress. *Acta Biochim. Polon*. **55**, 457–471 (2008). 18787711

[37] Naeem, M. et al. Radiation processed carrageenan improves plant growth, physiological activities, and alkaloids production in *Catharanthus roseus* L.* Adv. Bot.*** 2015**, 150474. 10.1155/2015/150474 (2015).

[38] El-Beltagi, H. S. et al. Potentiating biosynthesis of alkaloids and polyphenolic substances in *Catharanthus roseus* plant using ĸ-carrageenan. *Molecules* **28** (8), 3642. 10.3390/molecules28083642 (2023). 37110876 10.3390/molecules28083642 PMC10143362

[39] Mousavi, E. A., Kalantari, K. M., Nasibi, F. & Oloumi, H. Effects of Carrageenan as elicitor to stimulate defense responses of Basil against *Cuscuta campestris* Yunck. *Acta Bot. Croat*. **77**, 62–69. 10.2478/botcro-2018-0005 (2018).

[40] Jiang, J. L., Zhang, W. Z., Ni, W. X. & Shao, J. W. Insight on structure-property relationships of Carrageenan from marine red Algal: A review. *Carbohydr. Polym.* **257**, 117642. 10.1016/j.carbpol.2021.117642 (2021). 33541666 10.1016/j.carbpol.2021.117642

[41] Scherer, H. Sulphur in crop production—Invited paper. *Eur. J. Agron.* **14**, 81–111. 10.1016/S1161-0301(00)00082-4 (2001).

[42] Singh, V. & Rathore, S. S. Effect of applied potassium and sulphur on yield, oil content and their uptake by linseed. *J. Potassium Res.* **10**, 407–410 (1994).

[43] Umar, S., Debnath, G. & Bansal, S. K. Groundnut pod yield and leaf spot disease as affected by potassium and sulphur nutrition. *Indian J. Plant. Physiol.* **2**, 59–64 (1997).

[44] Ahl, S. A. & Mahmoud, A. A. H.A.H. Effect of zinc and/or iron foliar application on growth and essential oil of sweet Basil (*Ocimum Basilicum* L.) under salt stress. *Ozean J. Appl. Sci.* **3** (1) (2010).

[45] Hashmi, N. et al. Depolymerized Carrageenan ameliorates growth, physiological attributes, essential oil yield and active constituents of *Foeniculum vulgare* mill. *Carbohydr. Polym.* **90** (1), 407–412. 10.1016/j.carbpol.2012.05.058 (2012). 24751059 10.1016/j.carbpol.2012.05.058

[46] Singh, M., Khan, M. M. A., Uddin, M., Naeem, M. & Qureshi, M. I. Proliferating effect of radiolytically depolymerized Carrageenan on physiological attributes, plant water relation parameters, essential oil production and active constituents of *Cymbopogon flexuosus* Steud. Under drought stress. *PLoS ONE*. **12** (7), e0180129. 10.1371/journal.pone.0180129 (2017). 28708833 10.1371/journal.pone.0180129 PMC5510827

[47] Lachowicz, K. J. et al. Characteristics of plants and plant extracts from five of Basil (*Ocimum Basilicum* L.) grown in Australia. *J. Agric. Food Chem.* **45** (7), 2660–2665 (1997).

[48] Chagonda, L. S., Makanda, C. D. & Chalchat, J. C. The essential oils of *Ocimum canum* sims (basilic camphor) and *Ocimum urticifolia* Roth from Zimbabwe. *Flavour. Fragr. J.* **15** (1), 23–26 (2000).

[49] Hosseini, A., Zare Mehrjerdi, M. & Aliniaeifard, S. Alteration of bioactive compounds in two varieties of Basil (*Ocimum Basilicum*) grown under different light spectra. *J. Essent. Oil Bear. Plants* **21** (4), 913–923. 10.1080/0972060X.2018.1526126 (2018).

[50] Dzida, K. & Pol, S. Biological value and essential oil content in sweet Basil (*Ocimum Basilicum* L.) depending on calcium fertilization and cultivar. *Acta Sci. Polon. Hort. Cult.* **9** (4), 153–161 (2010).

[51] Omidbaigi, R., Hassani, A. & Sefidkon, F. Essential oil content and composition of sweet Basil (*Ocimum basilicum*) at different irrigation regimes. *J. Essent. Oil Bear. Plants*. **6** (2), 104–108. 10.1080/0972-060X.2003.10643335 (2003).

[52] Lenti, L. et al. A rapid procedure for the simultaneous determination of Eugenol, Linalool and fatty acid composition in Basil leaves. *Foods* **11** (21), 3315. 10.3390/foods11213315 (2022). 36359928 10.3390/foods11213315 PMC9659207

[53] Zheljazkov, V. D., Cantrell, C. L., Evans, W. B., Ebelhar, M. W. & Coker, C. Yield and composition of *Ocimum basilicum* L. and *Ocimum sanctum* L. grown at four locations. *Hortic. Sci.* **43** (3), 737–741. 10.21273/HORTSCI.43.3.737 (2008).

[54] Wilczek, R. et al. Effect of radiation-modified kappa-carrageenan on the morpho-agronomic characteristics of *Mungbean Vigna* radiata. *Philippine J. Sci.* **149** (S1), 135–143 (2019).

[55] Lyca, M., Trangia, C. & Macusi, E. S. Comparative performance of irradiated and non- irradiated carrageenan-based foliar fertilizers on the growth, yield, and pest incidence of Pechay (*Brassica rapa* L). *Davao Res. J.* **14** (1), 94–107. 10.59120/drj.v14i1.19 (2003).

